# Knowledge, Attitudes, and Practices Related to Colorectal Cancer's Prevention and Early Detection Among Older Adults in Kuwait: A Cross-Sectional Study

**DOI:** 10.7759/cureus.62323

**Published:** 2024-06-13

**Authors:** Abdullah M Alharran, Retaj S Aljuma, Aminah S Aljasmi, Mohammad F Al-Mutairi, Danah F M Alenezi, Yaqoub Y Alenezi, Hajar N Alajmi, Abdulbadih R Saad, Ahmed A Jaradat

**Affiliations:** 1 Department of Medicine and Surgery, College of Medicine, Arabian Gulf University, Manama, BHR; 2 Department of Family and Community Medicine, Arabian Gulf University, Manama, BHR

**Keywords:** colorectal, screening, attitudes, practices, knowledge

## Abstract

Background: Worldwide, colorectal cancer is one of the significant public health concerns that imposes a substantial risk of morbidity and mortality. Early detection and management of colorectal cancer are necessary to improve the patient's prognosis and outcomes. Hence, several guidelines recommend screening patients at risk of colorectal cancer periodically. Patients' knowledge and attitudes toward screening measures influence their compliance with the guidelines. This study aimed to determine knowledge, attitudes, and practices related to prevention and early detection among older adults in Kuwait.

Methods: A cross-sectional study was conducted among Kuwaiti adults aged between 45 and 75 years attending the outpatient department in Kuwait's seven major hospitals. A convenience sampling technique was used to recruit the participants. A self-administered questionnaire consisted of four parts: sociodemographic and baseline characteristics, knowledge of colorectal cancer screening, attitudes toward colorectal cancer screening, and practices toward colorectal cancer screening.

Results: A total of 570 Kuwaiti patients were included, and half of them were males (n = 285). Most patients were aged between 45 and 50 years (53.8%), had a secondary school certificate (n = 357, 62.6%), and were unmarried (n = 419, 73.5%). Low levels of knowledge (<50%) and practices (<30%) toward colorectal cancer screening were seen among the participants. Statistically significant correlations were found between patients’ knowledge about colorectal cancer screening and their attitudes (r = 0.317, P < 0.001) and practices (r = 0.330, P < 0.001). In addition, a moderately significant association was found between patients’ attitudes and practices toward colorectal cancer screening.

Conclusion: The study found that despite positive attitudes, Kuwaiti patients have low levels of knowledge and practice regarding colorectal cancer screening. This suggests a need for targeted, culturally sensitive educational programs and national campaigns to improve screening rates and address knowledge gaps.

## Introduction

Colorectal cancer (CRC) remains a significant public health concern globally, accounting for a substantial risk of morbidity and mortality. It is the third most prevalent cancer and the fourth most common cause of death, following lung, stomach, and liver cancer [1]. In recent years, a significant rise of 60% has been predicted for CRC, with an estimated 1.1 million deaths. The rate at which CRC occurs varies significantly around the world, with more cases reported in developed countries than in developing ones. Almost 55% of cases have been reported from developed regions of the world [2].

Colorectal adenocarcinoma is the most common type of CRC, characterized by the presence of malignant glandular epithelial cells [3]. Colorectal adenocarcinoma typically originates from benign adenomatous polyps in the colorectal epithelium, with the majority of cases arising from these precursor lesions [4,5]. This process involves the infiltration and subsequent destruction of adjacent normal tissues. As it progresses, colorectal adenocarcinoma invades the surrounding anatomical structures and metastasizes to distant organs like the liver, brain, and bone [6].

Various factors, including the cancer site, size, and stage, influence the symptoms of CRC [7]. Bleeding and altered bowel habits are the most frequent, with pain and bowel obstruction also common [7-10]. These symptoms can vary based on the location and stage of the cancer, with pain being more associated with colon cancer and fecal admixtures with rectal cancer [7]. Furthermore, patients can misinterpret their symptoms as benign intestinal issues without ever realizing the actual nature of the disease [11].

CRC-associated mortality is highly preventable, as detection of the disease in its early stages increases survival rates [12]. Because of this, knowledge about CRC can help with early diagnosis and prompt intervention, which can significantly improve patient outcomes. Therefore, a range of screening methods exist for CRC, including non-invasive approaches like fecal occult blood tests (FOBT), fecal immunohistochemistry (FIT), and stool DNA tests, as well as invasive techniques such as flexible sigmoidoscopy and colonoscopy [13]. Non-invasive methods, in particular, have shown promise in improving screening rates and reducing resource burden [14]. However, current stool-based tests have limitations, including high false-positive rates and low sensitivity [15]. Despite these challenges, the development of alternative non-invasive tests, such as those based on urine, exhaled breath, and blood, may offer improved diagnostic performance and patient experience [15]. Further research is needed to validate the diagnostic accuracy and generalizability of these new tests [16]. Since early CRC stages are asymptomatic, screening at this stage is crucial [17].

A number of researchers have evaluated the level of awareness and knowledge of colorectal screening among populations in numerous countries. In Bahrain, Nasaif and Al Qallaf reported a moderate knowledge level among adults aged 25 years and above [17]. In Saudi Arabia, studies by Alshammari et al. found that knowledge, attitude, and practices regarding CRC are generally low, with significant gaps in awareness of risk factors and screening methods among the population [18]. Tfaily et al. highlighted varying awareness levels across the Middle East and North Africa, noting particularly low knowledge in Lebanon and the United Arab Emirates [19]. Several studies indicate high levels of CRC knowledge among the general population. In China, 80% of participants demonstrated sufficient knowledge [20], while in Pakistan, 66.6% of university students showed awareness of CRC and its risk factors [21]. However, there was poor recognition of warning signs, with only 49% identifying them correctly. Despite this, positive attitudes prevailed, with over 90% believing in the importance of early diagnosis and around 80% acknowledging the significance of lifestyle modifications and regular screenings [21].

The American Cancer Society (ACS) recommends regular CRC screenings for individuals aged 45 to 75 years with average risk [22]. This includes those without a personal or familial history of CRC, inflammatory bowel disease, or radiation therapy in the abdomen or pelvic region [22]. However, individuals at increased risk, such as those with a personal or family history of advanced adenomas or CRC, should undergo more frequent or earlier testing [23]. The ACS's guideline update to lower the screening start age to 45 years would impact an estimated 19 million people in the US [24].

Research indicates that lack of public awareness, financial obstacles, and insufficient understanding of CRC screening protocols are the main contributors to CRC mortality [25,26]. As a result, understanding the level of knowledge, attitudes, and beliefs of a specific population is critical in creating targeted messages and educational materials aimed at promoting screening participation.

This study aims to evaluate the knowledge, attitudes, and practices among Kuwaiti adults attending Kuwait public hospitals to improve the utilization of CRC screening programs. The research will address the level and determinants of these factors and explore their association, focusing on how demographic variables influence knowledge, attitudes, and practices.

## Materials and methods

Study design

This study adopted a descriptive cross-sectional study design.

Study population

The study population of the present study included adult Kuwaiti citizens aged between 45 and 75 years attending the outpatient department in the seven major hospitals in Kuwait. These were Al-Adan Hospital, Al-Jahra Hospital, Al-Farwaniya Hospital, Sheikh Jaber Al-Ahmad Al-Sabah Hospital, Mubarak Al-Kabeer Hospital, Amiri Hospital, and Al-Sabah Hospital.

Selection criteria (inclusion and exclusion criteria)

This study analyzed Kuwaiti citizens aged 45 to 75 years who consented to participate. Exclusions comprised individuals who declined participation, were severely ill, served as healthcare providers, non-Kuwaiti citizens, and those who did not speak Arabic or English.

Sample size

The following equation was used for calculating the sample size: n = [Za/2^2 * P * (1-P)]/E^2. Where Za/2 corresponds to the standard variable at the (1-a)% confidence level (with a value of 1.96 for a 95% confidence interval). Based on earlier research [27,28], about 70% of the population was knowledgeable about CRC, and P stands for the percentage of that population that is knowledgeable about it. E stands for the 5% margin of error, and the sample size that was calculated was 323. The confidence interval level was 95%, and the margin of error was 0.05. To account for non-respondents, we included 570 participants in the sample.

Sampling methodology and data collection resources

A convenience sampling technique was used to recruit the participants. Data and resources were collected at the seven major hospitals in Kuwait. Patients’ data were gathered from outpatient department patients. Self-administered surveys were distributed in hard copies.

Study instrument

The self-administered questionnaire, adapted from Alshammari et al. [18], comprised four sections. The first gathered sociodemographic data including gender, age, nationality, marital status, and education level. Following this, the second section evaluated patients' knowledge with 11 questions. The third section gauged attitudes toward CRC screening via a Likert scale, spanning from "strongly agree" to "strongly disagree" across six questions. Finally, the fourth part consisted of two questions concerning practices toward CRC screening programs. Regarding knowledge assessment, correct answers were scored 1, while incorrect ones received 0. Scores were categorized into good (median score and above) and bad (below median score) knowledge. Attitudes were measured on a five-point Likert scale, with scores categorized as moderate (60-79%), high (80% or above), and low (less than 60%) based on Bloom’s cut-off point criteria as per previous research [18].

Statistical analysis

The data were collected, tested, and coded by the researchers. IBM SPSS version 26 (IBM Corp., Armonk, NY) was used for data entry, management, analysis, code checking, and data cleaning to remove entry errors incorporated in the database and ensure the consistency of responses. Frequencies and percentages were computed for categorical variables such as gender, age group, educational level, and marital status, while means and standard deviations were computed for continuous variables. A t-test and a correlation test were used to compare the groups. In all tests, a p-value of less than 0.05 was considered statistically significant.

Ethical consideration

The study received ethical approval from the Research and Ethics Committee, College of Medicine and Medical Sciences, Arabian Gulf University. A written consent form was obtained from all patients. The administrations of the seven major hospitals also received approval. Furthermore, no patient-identifying information was obtained.

## Results

Socio-demographic characteristics of the participants

A total of 570 Kuwaiti patients were included in the present study. Half of the participants were males (n = 285). Most patients aged between 45 and 50 years (53.8%) had a secondary school certificate (n = 357, 62.6%) and were unmarried (n = 419, 73.5%) (Table 1).

**Table 1:** Sociodemographic characteristics of the participants.

Variable		N (%)
Age group	<50 years	304 (53.3)
	50-65 years	220 (38.6)
	>65 years	46 (8.1)
Sex	Male	285 (50)
	Female	285 (50)
Educational level	Primary	110 (19.3)
	Secondary	357 (62.6)
	College	72 (12.6)
	Postgraduate	31 (5.4)
Marital status	Single	229 (40.2)
	Married	151 (26.5)
	Divorced	35 (6.1)
	Widowed	155 (27.2)
Heard about colorectal cancer screening	Yes	370 (64.9)
	No	200 (35.1)

**Figure 1:**
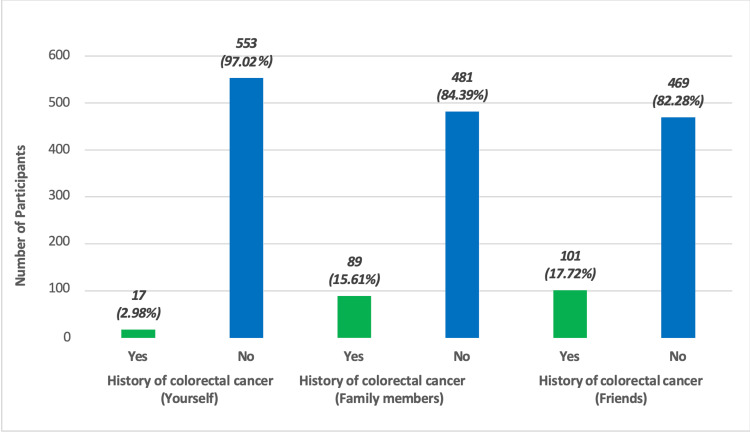
History of colorectal cancer among patients.

From the sample, 3% had CRC, 15.6% had a family member with CRC, and 17.7% had a friend with CRC (Figure 1).

Knowledge of participants about colorectal cancer screening

Nearly half of the participants were aware of CRC screening (n = 268, 47%), showed a willingness to undergo CRC screening even without experiencing any symptoms (n = 316, 55.4%), would agree to do colonoscopy if suggested by their doctor (n = 295, 51.8%), and reported planning to undergo CRC screening in the future (n = 268, 47%). Nonetheless, most of the participants were not aware of colorectal carcinoma screening tools (n = 343, 60.2%) and did not undergo CRC screening in Kuwait (n = 480; 84.2%) (Table 2).

**Table 2:** Knowledge about colorectal cancer screening.

Statement	Yes, N (%)	No, N (%)
Aware of colorectal cancer screening	268 (47)	302 (53)
Colorectal cancer can be prevented	389 (68.2)	181 (31.8)
Aware of any screening methods for colorectal cancer, such as home stool cards, sigmoidoscopy, or colonoscopy	227 (39.8)	343 (60.2)
Undertook colorectal cancer screening in Kuwait	90 (15.8)	480 (84.2)
Willing to undergo screening for colorectal cancer even without experiencing any symptoms	316 (55.4)	254 (44.6)
Agree to undergo a colon examination by inserting an endoscope into the anus	295 (51.8)	275 (48.2)
Agree to do this procedure in Kuwait	333 (58.4)	237 (41.6)
Plan to undergo colorectal cancer screening in the future	268 (47)	302 (53)
In your opinion, early screening decreases the incidence and mortality of colorectal cancer	508 (89.1)	62 (10.9)

**Figure 2:**
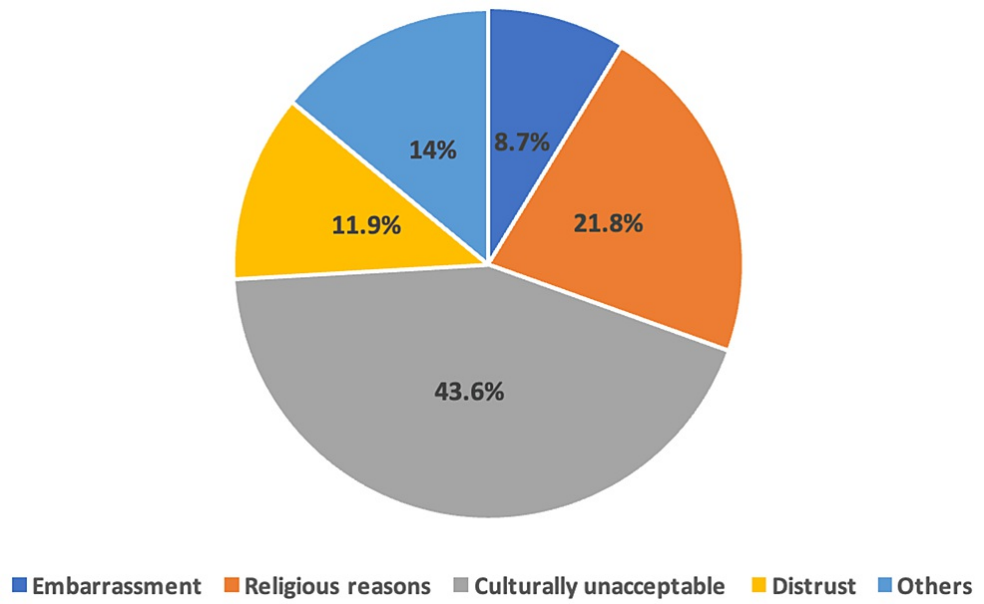
Reasons for not undergoing a colonoscopy among the participants.

**Figure 3:**
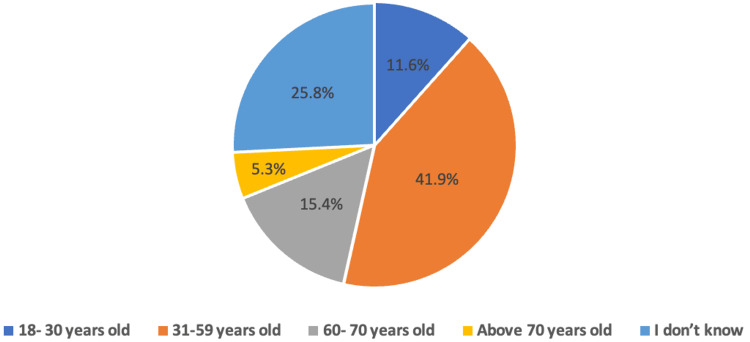
The recommended age of screening for colorectal cancer according to the participants.

The primary reason for not undergoing CRC screening was cultural unacceptability (43.6%) (Figure 2). Additionally, most participants (41.9%) believed that the recommended age for screening is between 31 and 59 years (Figure 3).

Attitudes toward colorectal cancer screening

Most participants agreed or strongly agreed that regular checkups for CRC are important even in the absence of a family history of the disease (74.8%), that CRC can be prevented if detected early (86.0%), and that CRC can be prevented by adopting a healthy diet (66.5%). Less than half of the patients stated that they used traditional medicine to treat their health issues before trying modern medicine (Table 3).

**Table 3:** Attitudes about colorectal cancer screening.

Statement	Strongly agree	Agree	Neutral	Disagree	Strongly disagree
Even if I do not have a family history of colorectal cancer, it is important to be checked regularly	241 (42.3)	185 (32.5)	101 (17.7)	39 (6.8)	4 (0.7)
Colorectal cancer can be cured if it is detected early through cancer screening	274 (48.1)	216 (37.9)	67 (11.8)	9 (1.6)	4 (0.7)
I sometimes use traditional medicine to treat my health issues before trying modern medicine	109 (19.1)	151 (26.5)	147 (25.8)	117 (20.5)	46 (8.1)
Colorectal cancer can be prevented by eating a healthy diet	208 (36.5)	171 (30.0)	134 (23.5)	43 (7.5)	14 (2.5)
When I think about getting screened for colorectal cancer, I feel good about it	184 (32.3)	163 (28.6)	125 (21.9)	67 (11.8)	31 (5.4)
Most people are afraid of undertaking colorectal cancer screening	216 (37.9)	212 (37.2)	96 (16.8)	32 (5.6)	14 (2.5)

Practices toward colorectal cancer screening

Most of the respondents did not perform checkups for CRC (n = 464, 81.4%) and did not think about undergoing screening for early detection of colon cancer (n = 382, 67%) (Table 4).

**Table 4:** Practices toward colorectal cancer screening.

Statement	Yes	No
Done early checkups for colorectal cancer	106 (18.6)	464 (81.4)
Thought about undergoing a screening for early detection of colon cancer	188 (33.0)	382 (67)

Correlation between patients’ knowledge, attitudes, and practices toward colorectal screening

Statistically significant correlations were found between patients’ knowledge about CRC screening and their attitudes (r = 0.317, P < 0.0001) and practices (r = 0.330, P < 0.0001). In addition, a moderately significant association was found between patients’ attitudes and practices toward CRC screening (Table 5).

**Table 5:** Correlation between patient’s knowledge, attitudes, and practices.

		Attitude	Practices
Knowledge	Pearson correlation	0.317	0.330
	Sig. (2-tailed)	<0.001	<0.001
Attitude	Pearson correlation		0.409
	Sig. (2-tailed)		<0.001

Knowledge, attitudes, and practices toward colorectal cancer screening and sociodemographic

As shown in Table 6, female participants (P = 0.030) and participants aged less than 50 years (P = 0.001) had better practices toward CRC screening. No other significant associations were found between sociodemographics and participants’ knowledge, attitudes, and practices toward CRC screening.

**Table 6:** Association between sociodemographic characteristics of participants and their knowledge, attitudes, and practices related to colorectal cancer screening.

		Knowledge		Attitude		Practices	
		Mean±SD	P-value	Mean±SD	P-value	Mean±SD	P-value
Sex	Males	1.36±0.19	0.214	2.09±0.64	0.986	1.71±0.38	0.030
	Females	1.38±0.18		2.08±0.55		1.78±0.35	
Age group	<50 years	1.37±0.17	0.188	2.13±0.56	0.197	1.79±0.33	0.001
	50-65 years	1.37±0.2		2.04±0.62		1.7±0.39	
	>65 years	1.32±0.19		2.01±0.7		1.62±0.41	
Marital status	Single	1.36±0.18	0.560	2.06±0.6	0.299	1.76±0.36	0.479
	Married	1.36±0.19		2.05±0.58		1.76±0.36	
	Divorced	1.38±0.14		2.23±0.66		1.7±0.35	
	Widowed	1.38±0.2		2.12±0.59		1.71±0.39	
Education	Primary	1.37±0.2	0.407	2.2±0.63	0.082	1.79±0.34	0.402
	Secondary	1.37±0.19		2.05±0.58		1.72±0.37	
	College	1.35±0.19		2.06±0.6		1.76±0.39	
	Postgraduate	1.42±0.16		2.2±0.63		1.77±0.34	

## Discussion

The present study aimed to determine Kuwaiti patients' knowledge, attitudes, and practices toward CRC screening. The results showed that Kuwaiti people had low knowledge and practice levels but positive attitudes regarding CRC screening. In addition, significant correlations were found between patients’ knowledge about CRC screening and their attitudes and practices (r = 0.330, P < 0.0001).

The findings of the present study, which indicate low levels of knowledge and practice but positive attitudes toward CRC screening among Kuwaiti patients, highlight several critical issues. Firstly, the discrepancy between positive attitudes and low knowledge and practice suggests a potential gap in effective health education and communication strategies. While patients may recognize the importance of screening, they may lack access to or understanding of the necessary information to translate this recognition into action. This gap could be due to insufficient public health campaigns, limited outreach by healthcare providers, or cultural barriers that prevent the effective dissemination of information.

The significant correlation between knowledge and both attitudes and practices (r = 0.330, P < 0.0001) underscores the importance of educational interventions. Increasing knowledge about CRC screening could positively influence attitudes and encourage proactive screening practices. This suggests that targeted educational programs could be a key strategy for improving screening rates.

For instance, a study of 231 subjects conducted by Alshammari et al. found that most participants had low levels of knowledge about screening for CRC [18]. Another study conducted by Jarab et al. in Jordan revealed that most patients had insufficient knowledge about CRC screening [29]. A cross-sectional study among 1912 participants in Saudi Arabia showed comparable levels of knowledge about CRC and its preventive measures [30]. Alshammari et al.'s findings indicate that low knowledge levels are not unique to Kuwait, but are prevalent in other regions as well [18]. Jarab et al.'s study in Jordan and the cross-sectional study in Saudi Arabia both found insufficient knowledge among patients, emphasizing a widespread need for enhanced educational efforts across different populations [29,30]. These studies collectively suggest that improving knowledge through tailored education could be a universally beneficial approach.

In the Republic of Kazakhstan, Toleutayeva et al. found similarly low levels of knowledge among the general population [31], further supporting the argument that knowledge deficits are a common barrier to effective CRC screening worldwide. This consistency across diverse geographical and cultural contexts highlights the universal challenge of educating the public about cancer prevention and screening.

The finding that most participants did not recognize the recommended age for colorectal carcinoma screening and were unaware of its methods is significant. This highlights a critical gap in public health education and awareness. The lack of knowledge about screening age and methods can lead to delays in diagnosis and treatment, adversely affecting patient outcomes. Imran et al. (2023) corroborated this finding in Saudi Arabia, where more than half of the patients could not identify the ideal age for screening [32], suggesting that this issue is not isolated but may be widespread.

Furthermore, the surprising revelation that healthcare professionals, including primary care physicians, also exhibit low levels of knowledge about CRC screening programs underscores a systemic problem. As reported by multiple studies [33-36], the inadequacy of knowledge among healthcare providers could impede effective patient education and referral for screening, exacerbating the issue. This suggests a need for enhanced training and continuous education programs for healthcare professionals to ensure they are well-informed about current screening guidelines and practices.

Our study's findings diverge from Jarab et al.'s 2023 paper [29] by showing no significant association between patients' knowledge about CRC screening and their sociodemographic characteristics. This lack of association suggests that factors beyond demographics influence knowledge levels. Possible explanations include variations in access to health education, differences in healthcare system interactions, or disparities in communication between healthcare providers and patients. These factors might overshadow the influence of age, personal or family medical history, or professional background on patients' knowledge.

Despite low knowledge levels, the majority of respondents displayed positive attitudes toward CRC screening. This finding indicates that, while patients may lack detailed information, they still recognize the importance of screening. This could be due to limited outreach by healthcare providers or cultural barriers that prevent the effective dissemination of information. The contrast with studies reporting low attitudes, such as the one by Colón-López et al. [37], may be attributed to methodological differences, such as sample size and composition. For instance, including older patients who have not previously been screened could skew results toward more negative attitudes. The positive attitudes toward screening and the strong correlation between knowledge, attitudes, and practices highlight the critical role of effective health education. Tailored educational programs that address specific barriers and leverage patients' generally positive attitudes could enhance CRC screening rates, ultimately improving early detection and patient outcomes.

Furthermore, our findings support existing literature that links knowledge, attitudes, and practices regarding CRC screening. The significant correlations observed suggest that increasing patients' knowledge could positively influence their attitudes and practices. This aligns with Alghamdi et al. and Zhang et al. [38,39], who reported that informed patients are more likely to engage in screening practices. These consistent associations across different studies underline the importance of educational interventions to improve screening uptake.

In line with this study's findings, which indicate that approximately 20% of patients underwent early checkups for CRC, it is noteworthy that this percentage is higher than previously reported rates in the literature, such as the 6.7% and 6.5% reported by Almadi et al. [26]. Interestingly, our study revealed that female participants and those under 50 years of age demonstrated better practices toward CRC screening, contradicting previous studies where sex and age were not significant determinants. Instead, our results align with those of Gabel et al. [40], who found health literacy to be a crucial determinant of knowledge and attitudes toward screening programs.

Previous literature has identified common reasons for not undergoing CRC screening, such as being asymptomatic, low awareness levels, and cultural factors, consistent with findings by Alshammari et al. and Koo et al. [18,41]. However, in our study, cultural unacceptance emerged as the primary reason for avoiding screening programs, underscoring the importance of considering cultural perspectives in educational interventions. Given the significant association between insufficient knowledge, attitudes, and practices toward CRC screening, our findings emphasize the necessity of educational programs and national campaigns tailored to address identified barriers and knowledge gaps, taking into account cultural determinants and patient perspectives. This reinforces the need for culturally sensitive interventions, as evidenced by the prevalence of culturally unaccepted tests as the primary deterrent to screening among our participants.

This study has several strengths. It is the first to evaluate the knowledge, attitudes, and practices regarding CRC screening among Kuwaiti patients, specifically in Kuwait, providing unique and localized insights. A relatively good sample size was obtained, enhancing the reliability of the findings.

However, this study also has some limitations. First, it included only Kuwaiti citizens, which limits the generalizability of the results to other populations within the region. Second, data were collected using a self-administered questionnaire, which introduces the potential for recall bias as respondents might not accurately remember or report their behaviors and attitudes. Additionally, a convenience sampling technique was employed, which may not fully represent the broader population, potentially impacting the study's overall representativeness.

## Conclusions

The present study revealed that Kuwaiti patients exhibit low levels of knowledge and practice regarding CRC screening, despite having positive attitudes. This highlights a crucial gap in effective health education and communication strategies. The significant correlation between knowledge and both attitudes and practices underscores the need for targeted educational interventions to improve screening rates. The findings also emphasize the importance of culturally sensitive educational programs and national campaigns to address identified barriers and knowledge gaps.
